# Caffeine Ingestion Increases Estimated Glycolytic Metabolism during Taekwondo Combat Simulation but Does Not Improve Performance or Parasympathetic Reactivation

**DOI:** 10.1371/journal.pone.0142078

**Published:** 2015-11-05

**Authors:** João Paulo Lopes-Silva, Jonatas Ferreira da Silva Santos, Braulio Henrique Magnani Branco, César Cavinato Cal Abad, Luana Farias de Oliveira, Irineu Loturco, Emerson Franchini

**Affiliations:** 1 Martial Arts and Combat Sports Research Group, School of Physical Education and Sport, University of São Paulo, São Paulo, Brazil; 2 NAR-Nucleus of High Performance in Sport, São Paulo, Brazil; 3 Laboratory of Applied Nutrition and Metabolism, School of Physical Education and Sport, University of São Paulo, São Paulo, Brazil; Victoria University, AUSTRALIA

## Abstract

**Objectives:**

The aim of this study was to evaluate the effect of caffeine ingestion on performance and estimated energy system contribution during simulated taekwondo combat and on post-exercise parasympathetic reactivation.

**Methods:**

Ten taekwondo athletes completed two experimental sessions separated by at least 48 hours. Athletes consumed a capsule containing either caffeine (5 mg∙kg^-1^) or placebo (cellulose) one hour before the combat simulation (3 rounds of 2 min separated by 1 min passive recovery), in a double-blind, randomized, repeated-measures crossover design. All simulated combat was filmed to quantify the time spent fighting in each round. Lactate concentration and rating of perceived exertion were measured before and after each round, while heart rate (HR) and the estimated contribution of the oxidative (W_AER_), ATP-PCr (W_PCR_), and glycolytic (W_[La-]_) systems were calculated during the combat simulation. Furthermore, parasympathetic reactivation after the combat simulation was evaluated through 1) taking absolute difference between the final HR observed at the end of third round and the HR recorded 60-s after (HRR_60s_), 2) taking the time constant of HR decay obtained by fitting the 6-min post-exercise HRR into a first-order exponential decay curve (HRR_τ_), or by 3) analyzing the first 30-s via logarithmic regression analysis (T30).

**Results:**

Caffeine ingestion increased estimated glycolytic energy contribution in relation to placebo (12.5 ± 1.7 kJ and 8.9 ± 1.2 kJ, P = 0.04). However, caffeine did not improve performance as measured by attack number (CAF: 26. 7 ± 1.9; PLA: 27.3 ± 2.1, P = 0.48) or attack time (CAF: 33.8 ± 1.9 s; PLA: 36.6 ± 4.5 s, P = 0.58). Similarly, RPE (CAF: 11.7 ± 0.4 a.u.; PLA: 11.5 ± 0.3 a.u., P = 0.62), HR (CAF: 170 ± 3.5 bpm; PLA: 174.2 bpm, P = 0.12), oxidative (CAF: 109.3 ± 4.5 kJ; PLA: 107.9 kJ, P = 0.61) and ATP-PCr energy contributions (CAF: 45.3 ± 3.4 kJ; PLA: 46.8 ± 3.6 kJ, P = 0.72) during the combat simulation were unaffected. Furthermore, T30 (CAF: 869.1 ± 323.2 s; PLA: 735.5 ± 232.2 s, P = 0.58), HRR_60s_ (CAF: 34 ± 8 bpm; PLA: 38 ± 9 bpm, P = 0.44), HRRτ (CAF: 182.9 ± 40.5 s, PLA: 160.3 ± 62.2 s, P = 0.23) and HRR_amp_ (CAF: 70.2 ± 17.4 bpm; PLA: 79.2 ± 17.4 bpm, P = 0.16) were not affected by caffeine ingestion.

**Conclusions:**

Caffeine ingestion increased the estimated glycolytic contribution during taekwondo combat simulation, but this did not result in any changes in performance, perceived exertion or parasympathetic reactivation.

## Introduction

Oxidative metabolism is the main metabolic pathway to provide energy during taekwondo combat [1], although the ability to maintain high-intensity actions via the anaerobic system is considered a further determining factor during a taekwondo match [2]. Campos et al. [1] demonstrated that high-intensity actions were maintained by ATP-PCr metabolism during taekwondo combat simulation, in which glycolytic metabolism has been considered important to perform numerous high-intensity actions [3]. Thus, nutritional strategies that could improve glycolytic metabolism, such as caffeine supplementation, could improve performance during simulated taekwondo combat. However, the effect of caffeine ingestion on energy cost during taekwondo combat is currently unexplored in the literature.

Rapid recovery between rounds is crucial to taekwondo success since official matches are composed of 3 rounds of 2 min with 1 min intervals [2]. Higher maximal aerobic power (VO_2max_) and, consequently, a faster parasympathetic reactivation during the intervals between rounds could help the athletes to improve taekwondo performance during either isolated (training) or consecutive combats (competition). Heart rate variability (HRV) and heart rate recovery (HRR) are non-invasive tools to provide information regarding the cardiac sympathetic and parasympathetic modulations during and after exercise [4]. Goldberger et al. [5] proposed a simple temporal time-varying parasympathetic index to evaluate parasympathetic reactivation after exercise, which is derived from the time-course of the root mean square of successive differences between R-R intervals (RMSSD) measured during successive 30-s segments (RMSSD_30s_). HRR can be assessed by the number of heart beats recovered within 60s after the cessation of exercise [6], fitting post-exercise HR recovery (HRR) to a first-order exponential decay [7, 8], and analyzing the time constant of 30s short-time recovery. Buchheit et al. [4] showed that all HRR indexes were significantly correlated with glycolytic metabolism during high-intensity exercise. Caffeine ingestion has previously been shown to impair parasympathetic reactivation [9], though it could be hypothesized that caffeine supplementation would prolong sympathetic activity that occurs during exercise, increasing both adrenergic factors and local metabolites (i.e. epinephrine, lactate) during recovery. Thus, caffeine may actual slow parasympathetic reactivation after combat simulation.

Caffeine (1,3,7 –trimethylxanthine) ingestion (3–9 mg∙kg^-1^) has been shown to significantly improve performance during endurance tasks (activities lasting greater than 30 min) [10], although its effect on short-term (< 30 min) high-intensity exercise is less clear [11]. Caffeine has been shown to increase anaerobic contribution [11] and reduce the rating of perceived exertion [12, 13] during exercise. However, little attention has been given to the effect of caffeine ingestion on combat sports, and only one study investigated its effect on taekwondo combat. Santos et al. [14] showed improved performance with 5 mg∙kg^-1^ caffeine supplementation on performance during simulated taekwondo combat. The results of this study suggested caffeine supplementation allowed individuals to maintain an increased exercise intensity during the latter rounds as indicated by increased blood lactate concentrations following the second and third rounds. However, no study to date has evaluated the effect of caffeine ingestion on the contribution of different energy systems during, or parasympathetic reactivation after, taekwondo combat simulation.

Therefore, the aim subsequent rmance feine on performance? You need to put this in order. My suggestion is below:easure following the second and thof this study was to evaluate the effect of caffeine ingestion on estimated energy systems contribution during simulated taekwondo combat and its subsequent effects on performance. Furthermore, we determined whether caffeine supplementation influenced parasympathetic indexes measured following the combat simulation. We hypothesized that caffeine supplementation would increase glycolytic metabolism, and thus lead to performance improvements, during the combat simulation. Furthermore, it was supposed that the higher glycolytic metabolism contribution with caffeine would delay cardiac autonomic recovery.

## Material and Methods

### Subjects

Ten male taekwondo athletes (age: 21 ± 4 years, body mass: 71.0 ± 12.9 kg, height: 1.80 ± 0.08 m), who were black belt holders with a minimum of 9 years’ experience took part in the present study. All of the subjects were active competitors in national and international championships. The athletes were at the competitive phase of their preparation and engaged in 10 sessions of training per week; approximately 1.5 h was dedicated to technical workouts, 12 h to tactical training and the remaining time to strength and flexibility (2h). The sample was representative of high-level male taekwondo athletes from all Olympic weight divisions: <58 kg (n = 3), 58–68 kg (n = 3), 68–70 kg (n = 2), >80 kg (n = 3). Prior to testing, the athletes were informed of the procedures, including the possible risks involved, and signed an informed consent form. This study was conducted in accordance with the International Ethical Guidelines and Declaration of Helsinki and all procedures were approved by the Ethics and Research Committee of the University of São Paulo.

*A priori* sample-size calculations, based on previously published data with similar participant characteristics [14], determined that 8 subjects would be necessary to detect a statistical difference given an estimated effect size of 0.5, a 1-β error probability of 0.8 and a *P* value significance level less than 0.05 for lactate concentration, which was the primary measurement used to estimate energy contribution during exercise. To account for athlete drop out, we aimed to recruit eleven participants. However, one participant was excluded from the study due to technical problems with the gas analyzer during collection and the athlete’s lack of time to repeat all procedures. Thus, statistical analysis was performed on ten participants.

### Experimental Design

**Fig 1:**
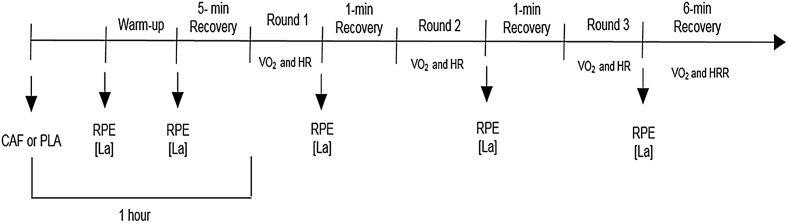
Experimental Design. CAF: Caffeine; PLA: Placebo; RPE: Rating of perceived exertion; [La^-^]: Plasma lactate concentration; HR: Heart rate; HRV: Heart rate variability; VO_2_: Oxygen uptake.

Athletes attended two sessions in which they were required to consume one capsule containing either 5 mg∙kg^-1^ of pure caffeine (CAF; Formula Ativa Pharmacy, São Paulo, São Paulo, Brazil) or cellulose (placebo; PLA) alongside 200 ml water, in a double-blind, randomized, repeated-measures crossover design (Fig 1). Immediately following combat simulation, athletes answered a questionnaire to evaluate any side effects (e.g., increased urine output, gastrointestinal problems, tachycardia, muscle soreness, or headache) experienced by participants during the combat simulation [15]. The two visits were separated by a 7-day washout period [14, 16, 17]. Following supplementation, athletes remained seated for 50-min and then performed a 5-min, self-selected warm-up. The self-selected warm-up was recorded and repeated in the subsequent experimental session. Thereafter, a 5-min rest was allowed and the combat was performed a full 1-hr after supplementation (Fig 1).

All tests were conducted at the same time of day (1 pm– 5 pm) to minimize circadian variance. To avoid the influence of diet on performance, athletes filled-out a 24-h food diary on the day preceding the first trial and were instructed to repeat the same diet on the day preceding the second test. Furthermore, during combat simulation the athletes did not ingest water or any food during the experiments. The athletes were also instructed not to ingest alcohol during the study and to refrain from consuming caffeine-containing substances (i.e., coffee, chocolate, and soft drinks) for 24-h before the experimental tests. Athletes were also required not to be performing any rapid weight loss in order to make weight during the period in which experiments were conducted. Habitual caffeine intake was assessed via a questionnaire with only two participants reporting a high-consumption level for caffeine (corresponding to more than six cups of coffee per day), while the remaining participants all reported a low-consumption level (less than two cups of coffee per day). The athletes performed the tests hydrated and fed, with the last meal ingested two hours before the start of the supplementation protocol. The temperature and relative humidity in CAF and PLA were 27.7°C, 13.4% and 29.5°C, 13.4%, respectively.

### Simulated Taekwondo Combat

Simulated combat was performed according to the World Taekwondo Federation’s (WTF) official rules and followed the Olympic weight categories. Briefly, each combat consisted of three 2-min rounds with 1-min intervals, and took place within an official area (8 x 8 m). A black belt in taekwondo who was blinded to the capsule content that the athletes had ingested refereed the matches. Although new taekwondo rules establish the use of electronic body protectors to protect the trunk of the athlete and at the same time allow more reliable and accurate score identification [18], we did not use this system because it would cause interference with the gas analyzer. The gas analyzer was placed on the athlete’s back and no attack to this area was allowed. Each athlete competed in a one-on-one match, and only one athlete was evaluated per match (CAF or PLA vs. non-supplemented athlete); the opponent was the same in both conditions. Furthermore, although coaches and others athletes were present during the experiments, no verbal encouragement was provided to the athletes. These procedures were similar to those used in studies analyzing karate [19] and taekwondo [1]. A video camera (Sony^®^ DCR-SX40, China) was used to record each combat for subsequent performance analysis.

### Physiological and Psychological Responses

Twenty-five microliters (μL) of blood was collected from the athletes’ earlobes to measure lactate concentration, analyzed by the electrochemical method, using a YSI Model 231 Lactate Analyzer (Yellow Sprints, US) which had been calibrated according to manufacturer instructions. The blood sample collections took place immediately before and after the warm-up, immediately after each round, and 1, 3 and 5-min after the last round.

Rating of perceived exertion (RPE) was measured immediately before and after the warm-up, and immediately before and after each round using the 6–20 Borg scale [20]. The subjects were asked to report RPE using cues derived from all sensations experienced during the exercise. Oxygen consumption was measured breath-by-breath during combat and rest using the K4b^2^ (Cosmed, Italy), which has previously been validated [21]. The athlete remained with the equipment during the entire combat and for 6-min after the activity ended. Mean oxygen consumption (VO_2MEAN_) for each round was calculated as the average VO_2_ during the entire time of the round. Since the gas analyzer uses telemetry, the start and finish of all combat phases were marked by one researcher who made these markers during all matches procedures. Thus, VO_2_ between rounds (VO_2INTERVAL_) also was recorded.

### Estimated Energy System Contribution

Estimates of the contributions of the oxidative, glycolytic and ATP-PCr systems during exercise were calculated using the measurements of oxygen consumption during activity, peak blood lactate concentration, and the fast phase of excess oxygen consumption after exercise (EPOC_FAST_). Oxidative metabolism (W_AER_) was estimated by subtracting resting VO_2_ (VO_2REST_; measured during a 5-min period before the warm-up) from VO_2MEAN_ during the rounds using the trapezoidal method [22]. The ATP-PCr system (W_PCR_) contribution was estimated using oxygen consumption during the interval between rounds and the EPOC_FAST_ after the third round [19, 22, 23]. In the present study, we fitted the kinetics of post-match oxygen consumption to bi- and mono-exponential models and, since the slow component of the bi-exponential model was negligible, post-match breath-by-breath VO_2_ data were fitted to a mono-exponential model and W_PCR_ was obtained by calculating the integral of the exponential part (Origin 6.0, Microcal, Massachusetts, USA). The contribution of the glycolytic system (W_[La_^-^_]_) was calculated using lactate concentration after combat, assuming that the accumulation of 1 mmol∙L^-1^ [La^-^] is equivalent to 3 ml O_2_ kg^-1^ of body mass [24]. The caloric quotient of 20.92 kJ [25] was used in all three different estimated energy systems. The delta change in lactate (Δ_[La_^-^_]_) was calculated as lactate concentration after each round, minus the lactate concentration at the beginning of the round.

Total metabolic work (W_TOTAL_) was calculated as the sum of the three estimated energy systems (W_AER_ + W_PCR_ + W_[La_^-^_]_. Additionally, the contribution of the three estimated energy systems was also expressed as a percentage relative to total energy expenditure.

### Heart Rate and post-exercise Heart Rate Recovery (HRR) assessment

Athletes remained in a seated position during the intervals between rounds. HR was measured continuously throughout the match simulation using a HR monitor (Polar S810i, Finland). Mean (HR_mean_) and peak (HR_peak_) HR were determined during each round. Parasympathetic function was assessed during the 6-min period after the third round. At the end of third round, all athletes immediately sat passively on a chair. Time between the end of exercise and sitting was less than 5-s. Particular attention to this detail was made because differences in body posture have been shown to result in different absolute HR recovery values. HRR was calculated by 1) taking the absolute difference between the final HR at the end of third round (mean of 5-s) and the HR recorded 60-s later (HRR_60s_: mean of 5-s), 2) taking the time constant of HR decay obtained by fitting the 6-min post-exercise HRR into a first-order exponential decay curve (HRR_τ_), or by 3) analyzing the first 30-s (from the 10^th^ to the 40^th^ seconds) via logarithmic regression analysis (T30) [4, 7].

### Time-varying vagal-related Heart Rate Variability (HRV) index

A progressive increase in the R-R interval is generally observed during the initial 5-min of HRR, although on shorter scales (i.e., 15-60-s), the curve is piecewise linear with superimposed oscillations. Thus, a time-varying vagal-related index (the root mean square of successive differences in the R-R intervals; RMSSD) was calculated for each of the subsequent 30-s segments of recovery (RMSSD_30s_). To smooth any transient outliers in the HRV plots (HRV vs recovery time), a median filter operation was performed in which each value was replaced with the median of the value as well as the preceding and following values. The first and last values were not median filtered [5].

### Video Analyses

The recorded images were analyzed using the Kinovea software (0.8.15), according to the criteria established by Santos et al. [14, 26]. Time-motion analyses were conducted in an attempt to calculate work:rest ratios for each match. The events were measured in tenths of a second, using the marking tool in the analysis software. Attack time (AT), stepping time (ST) and pause time (PT) were recorded separately for each round. The AT was the total time during which the athlete attacked or tried to attack his opponent. Specifically, we recorded the time elapsed between the moment that the athlete began to move his foot or hand in the direction of the opponent and the moment when the athlete finished the attack movement. The ST was defined as the total time in which there was no attempt to attack and the PT was characterized by time-outs determined by the referees.

The following indexes were calculated from these measurements: AT for each round; ST for each round; sum of AT for each round; sum of ST for each round; total attack number (AN) for each round; and average AT:ST ratio for each round [14, 26]. To verify the reliability of analysis, all matches were analyzed twice by the same researcher and a reliability of analyses was performed using an Intraclass Coefficient Correlation (ICC). The ICC revealed that the time-motion analyses conducted in this study was highly reliable for all 5 variables investigated, with values ranging from 0.95 to 0.99 (P < 0.05): attack time (ICC: 0.98; P < 0.01), total attack number for each round (ICC: 0.95; P <0.01), sum of attack time for each round (ICC: 0.99; P < 0.01), stepping time (ICC: 0.97; P < 0.01), sum of stepping time for each round (ICC: 0.99; P < 0.01).

### Statistical Analysis

The Kolmogorov-Smirnov test was applied to determine if the data met parametric assumptions (normality). The data were tested for homogeneity using Levene’s test. After confirmation of normality and homogeneity, the variables were compared using a two-way (condition and time of measurement) analysis of variance (ANOVA) with repeated measurements in the second factor (condition x time). Where necessary, a Bonferroni multiple comparison test was used in order to identify possible differences between conditions and time. When the assumption of sphericity was violated, as indicated by the Mauchley test, the critical value of F was adjusted using the Greenhouse-Geisser epsilon value. All indexes of HRR were compared using a Student’s t-test in both CAF and PLA. Effect sizes were calculated using eta squared (η^2^). A η^2^ of 0.2, 0.5 and 0.8 were considered as small, moderate and large effects [27]. The results of descriptive statistics are reported as the mean ± SD. Significance was defined as P < 0.05. All analyses were performed using SPSS software (version 17.0; IBM, Chicago, IL, USA).

## Results

### Success of the blinding and Pre-Test Recommendations

Of the ten athletes investigated, six athletes correctly distinguished which capsule had been ingested based on the effects they felt after taking it. CAF produced an increase in the sensations of tachycardia (20% of athletes), anxiety/nervousness (10% of athletes) and increases of vigor/activeness (60% of athletes). Furthermore, following pre-test instructions, all athletes confirmed that they did not drink alcohol, caffeine, or perform vigorous exercise during the 24 h before the tests.

### Physiological Measurements

The HR and oxygen consumption results are presented in Table 1. There was no main effect of condition for HR_mean_ (F_1,8_ = 3.02; P = 0.12; η^2^ = 0.27, [small]), but there was a main effect of round (F_2,16_ = 20.31, P = 0.01; η^2^ = 0.71, [moderate]), with lower values in the first round compared to the second (P = 0.01) and third (P = 0.01) rounds; similarly, there were lower values in the second round than in the third round (P = 0.04). However, there was no condition by round interaction (F_2,16_ = 0.46; P = 0.63; η^2^ = 0.05, [small]). There was no main effect of condition for HR_peak_ (F_1,8_ = 3.35; P = 0.10; η^2^ = 0.29, [small]), but there was a main effect of round (F_2,16_ = 12.15, P = 0.01; η^2^ = 0.60, [moderate]), with lower values in the first round compared to those in the second (P = 0.02) and third (P = 0.02) rounds, and lower values in the second round than in the third round (P = 0.05). However, there was no condition by round interaction (F_2,16_ = 2.32; P = 0.13; η^2^ = 0.22, [small]) (Table 1).

**Table 1:** Physiological responses during a taekwondo combat.

	Round 1		Round 2		Round 3	
	CAF	PLA	CAF	PLA	CAF	PLA
HR_mean_ (bpm)	170 ± 8^a^ ^,^ ^b^	167 ± 13^a^ ^,^ ^b^	175 ± 6^b^	173 ± 10^b^	178 ± 6	177 ± 10
HR_peak_ (bpm)	181 ± 10^a^ ^,^ ^b^	177 ± 12^a^ ^,^ ^b^	185 ± 7^b^	183 ± 10^b^	188 ± 8	188 ± 10
VO_2_ (ml.kg^-1^.min^-1^)	37.9 ± 4.2^a^ ^,^ ^b^	34.5 ± 5.5^a^ ^,^ ^b^	43.2 ± 5.9^b^	41.35 ± 3.6^b^	41.4 ± 5.1	38.4 ± 6.1

Data are reported as Mean ± SD. CAF: caffeine; PLA: placebo. HR_peak_: peak heart rate; HR_mean_: mean heart rate; VO_2mean_ = mean oxygen uptake.

^a^ different from round 2 (P < 0.05);

^b^ different from round 3 (P < 0.05).

There was no main effect of condition for VO_2mean_ (F_1,9_ = 4.33; P = 0.06; η^2^ = 0.32, [moderate]), but there was a main effect of round (F_2,18_ = 20.74; P = 0.01 η^2^ = 0.69, [large]), with lower values in the first round than in the second (P = 0.01) and third (P = 0.05) rounds, and lower values in the second compared to the third round (P = 0.05). However, there was no condition by round interaction (F_2,18_ = 0.65; P = 0.55; η^2^ = 0.06, [small]) (Table 1).

**Fig 2:**
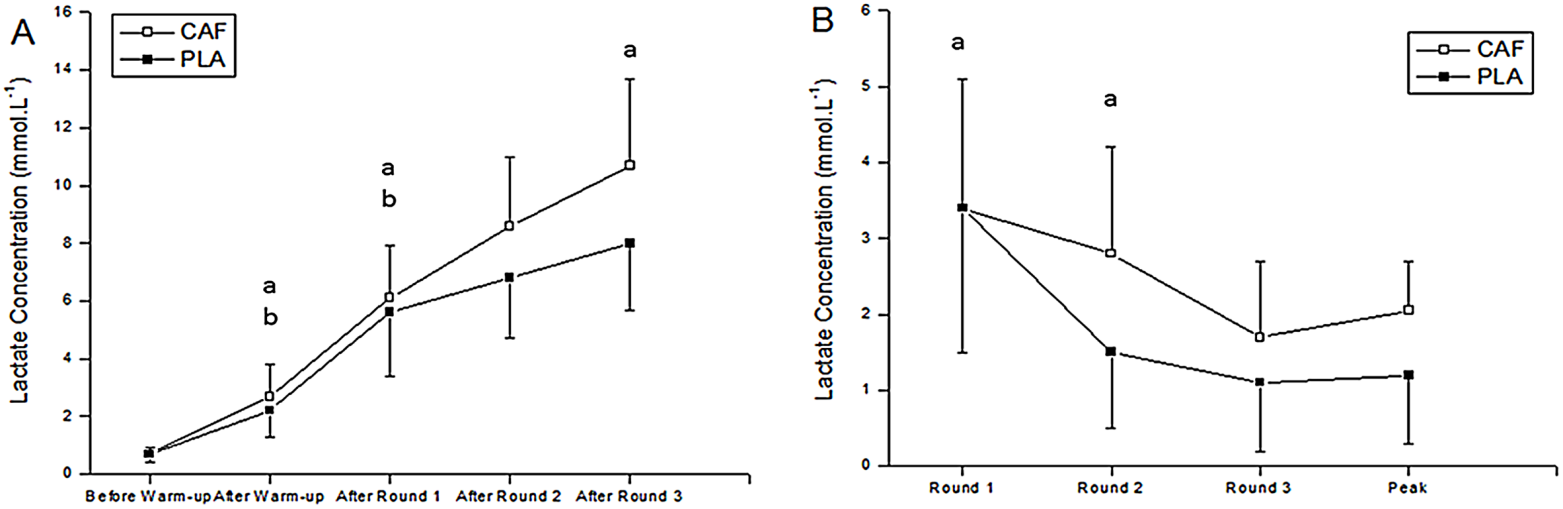
**a.** Peak blood lactate concentration before and after warm-up, and after rounds 1, 2 and 3; CAF = Caffeine; PLA = Placebo. ^a^significantly higher than the preceding value in PLA; ^b^significantly higher than the preceding value in CAF **Fig 2b.** Delta blood lactate concentration after the warm up minus blood lactate at the beginning of rounds 1, 2 and 3. ^a^significantly higher than round 3 (n = 10; values are mean ± SD).

There was a main effect of condition for [La^-1^]_peak_ (F_1,9_ = 8.90; P = 0.01; η^2^ = 0.5, [small]) with higher values in CAF compared to PLA (P = 0.03). Furthermore, [La^-1^]_peak_ increased significantly with time in all conditions (F_4,36_ = 120.27; P = 0.01; η^2^ = 0.93, [large]). Additionally, there was a condition by round interaction (F_4,36_ = 3.41; P < 0.01; η^2^ = 0.16, [small]), with values after the first round higher than before (P < 0.01) and after (P < 0.01) warm-up in CAF and PLA. Moreover, [La^-1^]_peak_ after the third round (P < 0.01) was significantly higher than following the second round in CAF (P < 0.01) (Fig 2a).

There was no main effect of condition for Δ [La^-^] (F_1,9_ = 2.64; P = 0.13; η^2^ = 0.22, [small]), but there was a main effect of round (F_2,18_ = 13.13; P = 0.01; η_p_^2^ = 0.6, [small]) with Δ [La^-^] being higher after the first round in relation to the second (P = 0.05) and third (P = 0.01) rounds. However, there was no condition by round interaction (F_2,18_ = 1.33; P = 0.28; η_p_^2^ = 0.12, [small]) (Fig 2b).

There was no main effect of condition on absolute estimated aerobic energy cost (F_1,9_ = 0.27, P = 0.61, η^2^ = 0.02, [small]), but there was a main effect of round (F_2,18_ = 13.81; P = 0.01; η^2^ = 0.60, [moderate]), with higher estimated absolute aerobic cost during the second round than the first (P = 0.01) and third rounds (P = 0.02). No condition by round interaction was found (F_2,18_ = 1.87; P = 0.18; η^2^ = 0.17, [small]). Similarly, there was no main effect of condition on the estimated relative aerobic cost (F_1,9_ = 0.12; P = 0.91; η^2^ = 0.01, [small]), but there was a main effect of round (F_2,18_ = 15.72; P = 0.01; η^2^ = 0.63, [moderate]), with higher estimated relative aerobic cost in the second round than in the first (P = 0.01) and third (P = 0.01) rounds. However, there was no condition by round interaction (F_2,18_ = 0.97; P = 0.39; η^2^ = 0.1, [small]) (Table 2).

**Table 2:** Estimated metabolic responses on the three rounds of taekwondo combat simulation in placebo and caffeine conditions.

	Round 1		Round 2		Round 3	
	CAF	PLA	CAF	PLA	CAF	PLA
Oxidative						
W_AER_ (kJ)	103.2 ± 11.8^b^	97.1 ± 7.8^b^	117.3 ± 15.2	116.8 ± 15.2	107.5 ± 20.8^b^	109.8 ± 16.4^b^
Relative (%)	66 ± 6^b^	63 ± 5^b^	66 ± 6	70 ± 3	65 ± 6^b^	64 ± 9^b^
ATP-PCr						
W _[PCR]_ (kJ)	37.8 ± 10.0	43.4 ± 7.4	43.1 ± 10.2	42.6 ± 8.7	55.3 ± 13.7^a^ ^b^	54.7 ± 24.2^a^ ^b^
Relative (%)	24 ± 4	27 ± 3	24 ± 3	26 ± 2	31 ± 4^a^ ^b^	33± 9^a^ ^b^
Glycolytic						
W_[La_^-^_]_ (kJ)	17.7 ± 9.94^c^	13.3 ± 6.9	11.8 ± 7.9^a^ ^c^	7.5 ± 5.5^a^	8.1 ± 4.3^a^ ^c^	5.8 ± 3.3^a^
Relative (%)	10 ± 5	9 ± 5	7 ± 4^a^	4 ± 3^a^	4 ± 2^a^	3 ± 2^a^
W_Total_ (kJ)	158.4 ± 22.7^b^	154.2 ± 17.0^b^	175.3 ± 26.3	163.9 ± 27.1	173.6 ± 28.0	167.6 ± 38.3

Data are reported as mean and standard deviation. CAF: Caffeine; PLA: Placebo. Waer = estimated oxidative energy, W_[La-]_ = estimated glycolytic energy, W_PCR_ = estimated ATP-PCr energy, W_Total_ = estimated total metabolic work (W_AER_ + W_[La-]_ + W_PCR_).

^a^Different than round 1;

^b^Different than round 2;

^c^Diferent than PLA condition.

There was a main effect of condition on estimated absolute glycolytic contribution (F_1,9_ = 5.60; P = 0.04; η^2^ = 0.38, [moderate]), with higher values in CAF compared to PLA (P = 0.04). Furthermore, there was a main effect of round (F_2,18_ = 10.47; P = 0.01; η^2^ = 0.53, [moderate]), with higher estimated absolute glycolytic contribution in the first round compared to the second (P = 0.04) and third (P = 0.01) rounds, but there was no condition by round interaction (F_2,18_ = 0.24; P = 0.78; η^2^ = 0.02, [small]). Similarly, for estimated relative glycolytic contribution there was a main effect of condition (F_1,9_ = 5.70; P = 0.04; η^2^ = 0.38, [moderate]), with higher values in CAF compared to PLA. Furthermore, there was a main effect of round (F_2,18_ = 13.10; P = 0.01; η^2^ = 0.60, [moderate]) with higher estimated relative glycolytic contribution in the first round compared to second (P = 0.02) and third (P = 0.01) rounds. However, there was no condition by round interaction (F_2,18_ = 1.32; P = 0.29; η^2^ = 0.12, [small]) (Table 2).

There was no main effect of condition on estimated absolute ATP-PCr contribution (F_1,9_ = 0.13; P = 0.72; η^2^ = 0.15, [small]), but there was a main effect of round on estimated absolute ATP-PCr contribution (F_2,18_ = 16.34; P = 0.01; η^2^ = 0.64, [moderate]), with higher values in the third round compared to the first (P = 0.01) and second (P = 0.01) rounds. However, there was no condition by round interaction (F_2,18_ = 0.62; P = 0.54; η^2^ = 0.06, [small]). Similarly, for estimated relative ATP-PCr contribution there was no main effect of condition (F_1,9_ = 1.83; P = 0.20; η^2^ = 0.16, [small]). However, there was a main effect of round for estimated relative ATP-PCr contribution (F_2,18_ = 19.41; P = 0.01; η^2^ = 0.68, [moderate]), with higher estimated relative ATP-PCr contribution in the third round when compared to the first (P = 0.01) and second (P = 0.02) rounds. In addition, no condition by round interaction was shown (F_2,18_ = 0.55; P = 0.94; η^2^ = 0.01, [small]) (Table 2).

There was no main effect of condition on estimated absolute energy expenditure (F_1,9_ = 2,24; P = 0.16; η^2^ = 0.20, [small]), but there was a main effect of round (F_2,18_ = 5.04; P = 0.01; η^2^ = 0.35, [moderate]), with higher estimated absolute energy expenditure in the second round compared to the first round (P = 0.01). Furthermore, there was no condition by round interaction (F_2.18_ = 0.46; P = 0.63; η^2^ = 0.04, [small]) (Table 2).

**Fig 3:**
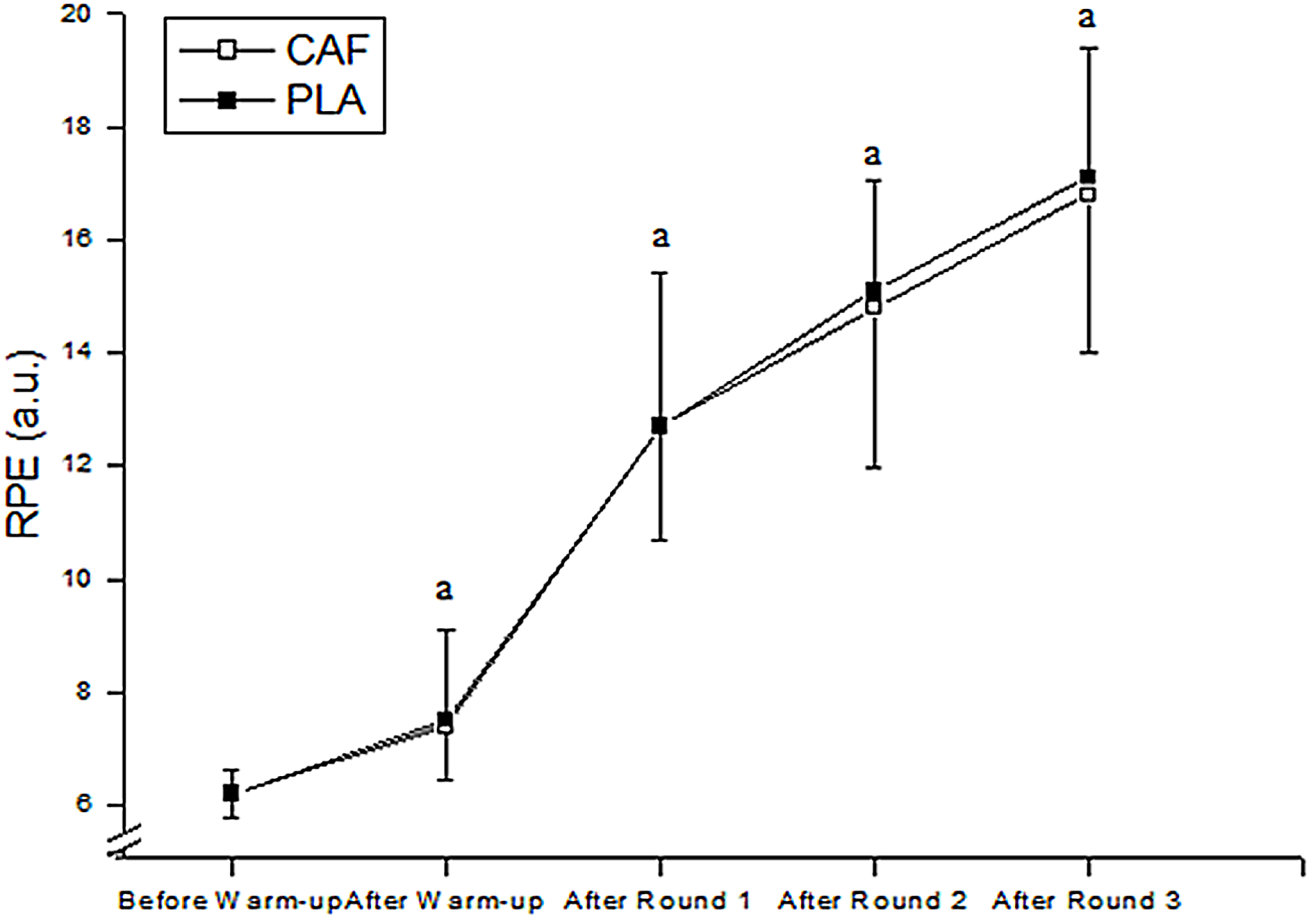
Rating of perceived exertion (RPE) before and after the warm-up and after each round in CAF and PLA. ^a^significantly higher than preceding values in CAF and PLA. CAF = Caffeine; PLA = Placebo.

There was no main effect of condition for RPE (F_1,9_ = 0.27; P = 0.62; η^2^ = 0.03, [small]), but there was a main effect of round (F_4,36_ = 105.82; P = 0.01; η^2^ = 0.92, [large]), with values increasing significantly throughout the combat in both CAF and PLA conditions (P = 0.01). However, there was no condition by round interaction (F_3,27_ = 1.24; P = 0.31; η^2^ = 0.12, [small]) (Fig 3).

### Time-Motion Analyses

Time-motion analyses are presented in Table 3. There was no main effect of condition (F_1,9_ = 0.31; P = 0.58; η^2^ = 0.03, [small]), round (F_2,18_ = 0.40; P = 0.67; η^2^ = 0.04, [small]) or condition by round interaction (F_2,18_ = 0.70; P = 0.50; η^2^ = 0.07, [small]) on AT. Similarly, there was no main effect of condition (F_1,9_ = 0.25; P = 0.62; η^2^ = 0.02, [small]), round (F_2,18_ = 3.17; P = 0.06; η^2^ = 0.26, [small]) or condition by round interaction (F_2,18_ = 0.49; P = 0.61; η^2^ = 0.05, [small]) for ST (Table 3).

**Table 3:** Attack Time, Stepping Time, total time of Attack Time, Stepping Time, ratio between Attack Time and Stepping Time, and ratio between summed time of the Attack Time + Stepping Time and Pause Time during a taekwondo combat simulation in both CAF and PLA conditions.

	Round 1		Round 2		Round 3	
	CAF	PLA	CAF	PLA	CAF	PLA
Attack Time (s)	1.3 ± 0.4	1.4 ± 0.8	1.3 ± 0.2	1.2 ± 0.3	1.2 ± 0.1	1.3 ± 0.2
Stepping Time (s)	3.0 ± 1.0	3.0 ± 0.8	3.3 ± 1.0	3.7 ± 1.1	3.8 ± 2.6	4.0 ± 1.6
Attack Time sum (s)	35.2 ± 8.8	45 ± 2,9	33.6 ± 6.5	32.0 ± 7.3	32.8 ± 8.1	33.0 ± 10.0
Stepping Time sum (s)	73.5 ± 18.1	76.0 ± 14.0	79.2 ± 11	79.7 ± 14.5	79.3 ± 9.7	78.5 ± 14.1
Attack Number (times)	28.0 ± 7.0	30.6 ± 7.0	25.1 ± 4.6	25.8 ± 7	27.2 ± 83	25.6 ± 8.5
Attack Time/Stepping Time ratio	0.5 ± 0.2	0.6 ± 0.5	0.4 ± 0.1	0.4 ± 0.2	0.4 ± 0.1	0.4 ± 0.18

CAF: Caffeine; PLA: Placebo.

There was no main effect of condition (F_1,9_ = 0.66; P = 0.43; η^2^ = 0.06, [small]), round (F_2,18_ = 2.45; P = 0.11; η^2^ = 0.21, [small]) or condition by time interaction (F_2,18_ = 2.20; P = 0.13; η^2^ = 0.19, [small] on the sum of attack time. Furthermore, there was no effect of condition (F_1,9_ = 0.10; P = 0.75; η^2^ = 0.01, [small]), round (F_2,18_ = 2.82; P = 0.08; η^2^ = 0.23, [small]) or condition by round interaction for the sum of stepping time (F_2,18_ = 0.27; P = 0.76; η^2^ = 0.02, [small]) (Table 3).

There was no effect of condition (F_1,9_ = 0.04; P = 0.83; η^2^ = 0.01, [small]), round (F_2,18_ = 3.05; P = 0.07; η^2^ = 0.25, [small]) or condition by time interaction on number of attacks (F_2,18_ = 1,27; P = 0.30; η^2^ = 0.12, [small]). Additionally, there was no effect of condition (F_1,9_ = 1.02; P = 0.33; η^2^ = 0.10, [small]), round (F_2,18_ = 0.38; P = 0.68; η^2^ = 0.04, [small]) or condition by time interaction on attack time/stepping time ratio (F_2,18_ = 1.18; P = 0.33; η^2^ = 0.11, [small]) (Table 3).

HRV indexes are presented in Table 4. There were no significant differences in HRR_60_ (t(8) = 1.17, P > 0.05, effect size = 0.38, [moderate], 95% CI = -3.96–12.1), HRR_τ_ (t(8) = -1.18, P > 0.05, effect size = 0.38, [moderate], 95% CI = -66.7–21.4), T_30_ (t(8) = -1.12, P > 0.05, effect size = 0.36, [moderate], 95% CI = -407.3–140.1) or HR_amp_ (t(8) = 1.57, P > 0.05, effect size = 0.48, [moderate], 95% CI = -4.2–22.3) between CAF and PLA.

**Table 4:** Heart rate variability indexes of parasympathetic reactivation in both CAF and PLA conditions.

	CAF	PLA
HRR_60s_, bpm	34 ± 8	38 ± 9
T_30_, s	869.1 ± 323.2	735.5 ± 232.2
HRR_τ_, s	182.9 ± 40.5	160.3 ± 62.2
HRR_amp_, bpm	70.2 ± 17.4	79.2 ± 17.4

HRR_60s_, number of heart rate beat recovered in 60-s after exercise cessation; T_30_, time constant of short-time heart rate recovery; HRR_τ_, time constant of HR during the 6-min recovery. CAF = Caffeine; PLA = Placebo.

**Fig 4:**
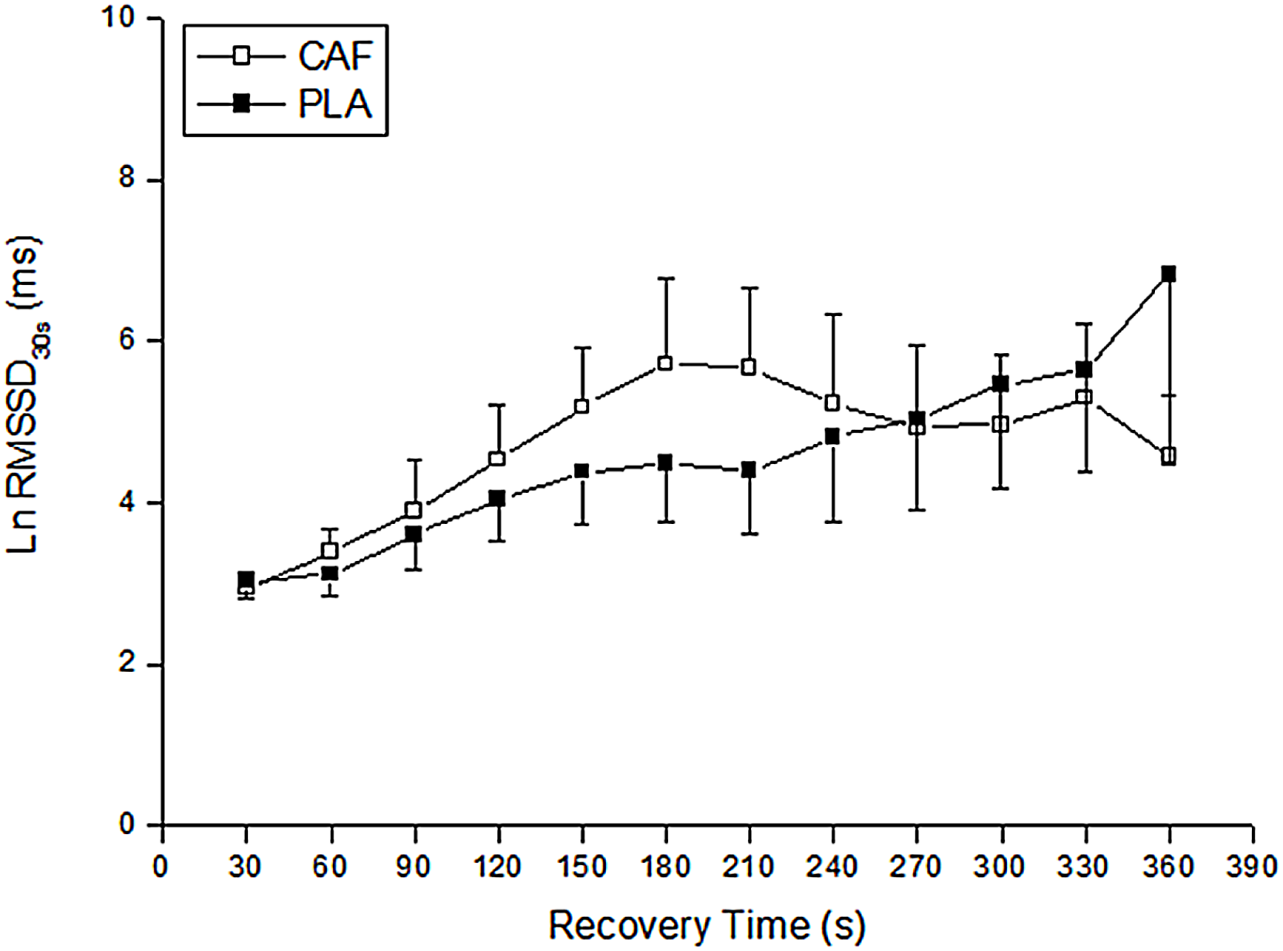
Root mean square of successive differences in the R-R intervals measured on successive 30-s segments (RMSSD_30s_) during the 6-min recovery period after the third round in both CAF and PLA.

The time course of the R-R interval after the third round is shown in Fig 4. There was no main effect of condition on RMSSD_30s_ (F_1,8_ = 0.67; P = 0.67; η^2^ = 0.07, [small]), time (F_11,88_ = 1.92; P = 0.16; η^2^ = 0.19, [small]) or a condition by time interaction (F_11,88_ = 1.96; P = 0.18; η^2^ = 0.19, [small]).

**Fig 5:**
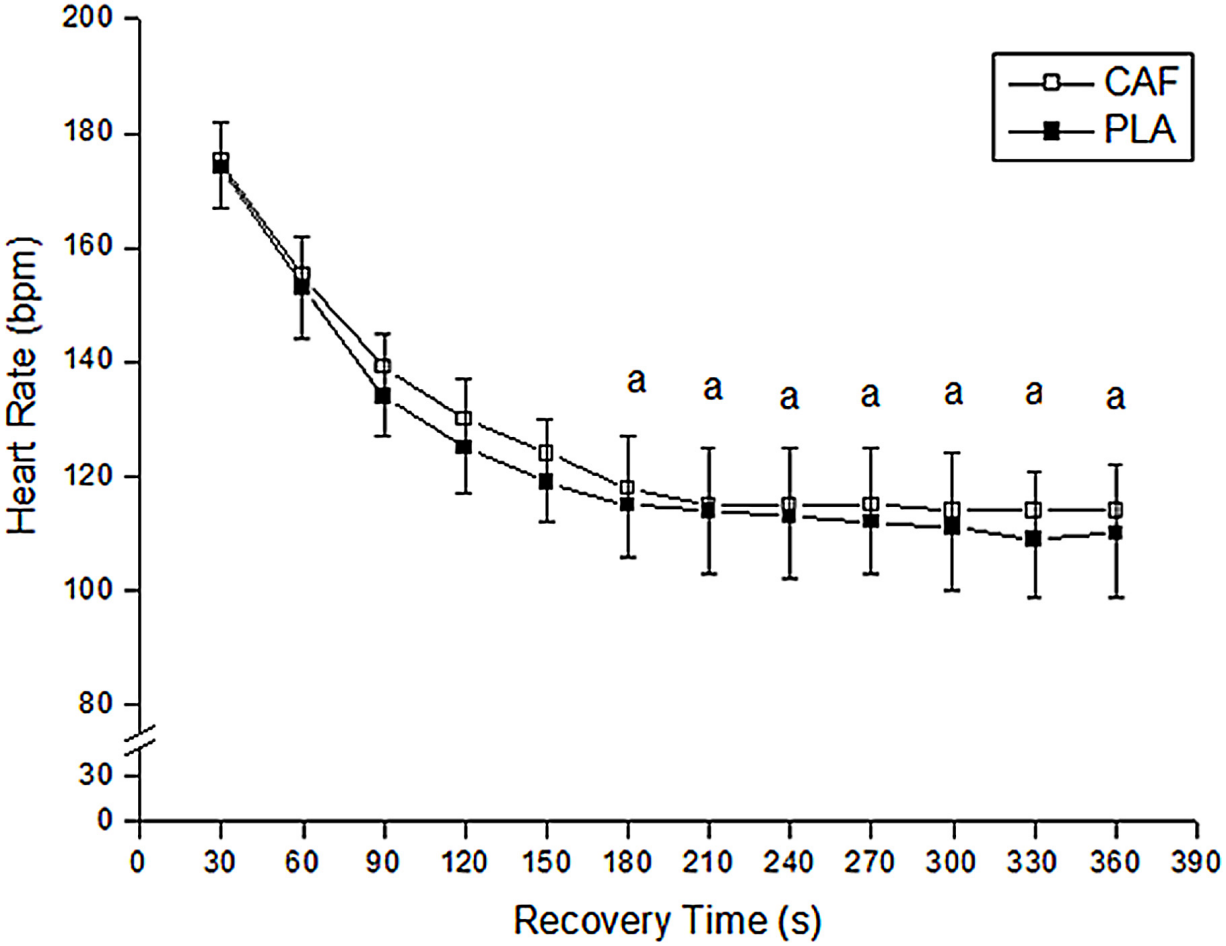
Heart Rate Recovery during the 6-min recovery period after the third round in both CAF and PLA. CAF = Caffeine; PLA = Placebo. ^a^significantly lower in relation to times 30, 60, 90, 120 and 150-s after the third round.

The time course of HRR after the third round is illustrated in Fig 5. There was no main effect of condition for HRR (F_1,8_ = 2.04; P > 0.05; η^2^ = 0.18, [small]). However, there was a main effect of time for HRR (F_11,99_ = 200.11; P < 0.05; η^2^ = 0.01, [small]), with values systematically higher until the 150s after the third round in relation to values after 180s of the recovery. Furthermore, there was no condition by time interaction (F_11,99_ = 1.63; P > 0.05; η^2^ = 0.15, [small]).

## Discussion

The main findings of the present study were that caffeine ingestion increases absolute and relative estimated glycolytic energy contribution during simulated taekwondo combat, although this did not translate into any improvements in performance. Furthermore, caffeine ingestion did not affect autonomic control after the combat.

In this study, caffeine supplementation did not change any time-motion performance variable during simulated taekwondo combat. Previous studies have reported benefits after caffeine ingestion on high-intensity exercise [10, 11, 16, 28], as well as during taekwondo combat simulation [14]. However, not all studies have shown a positive effect of caffeine with no performance changes shown during successive maximal cycling bouts [29], wrestling [30], and judo [17]. Similarly to our results, Lopes-Silva et al. [17] showed that caffeine ingestion did not improve performance (number of throws) during the Special Judo Fitness Test after a 5-day weight loss period. More recently, Felippe et al. [31] also reported that caffeine ingestion did not improve performance (number of throws) during the Special Judo Fitness Test. Conversely, Santos et al. [14] showed that caffeine supplementation reduced the sum of time-outs enforced by the referee in the first and second rounds of a taekwondo combat simulation. The reasons for these differences are not clear, but may be related to different performance protocols that used different durations, intensities and type of exercise. In the present study, we evaluated simulated taekwondo combat and during this activity performance is determined by technical, tactical, physiological and physical characteristics of the athletes [2]. Despite an increased estimated glycolytic contribution with caffeine this did not alter the pattern of technical actions during the simulation. However, in the study conducted by Santos et al. [14] the combats were performed between supplemented athletes (i.e. caffeine *versus* caffeine), which suggests that improvement in performance may have been caused by the fact that both athletes were supplemented. In contrast to the study of Santos et al. [14], athletes in the present study were evaluated individually (supplemented *versus* non-supplemented athletes). Thus, when only one athlete is assessed (i.e., the supplemented athlete) there was no improvement in performance variables following caffeine supplementation during simulated taekwondo combat.

Our results show that there was no significant difference in HR_mean_ and HR_peak_ during simulated taekwondo combat after caffeine ingestion. Our data corroborate with Santos et al. [14], who did not report significant differences in HR responses during taekwondo combat simulation after caffeine supplementation in relation to placebo. However, there was an increase in HR values over the course of the combat. This corroborates with other studies, which have shown an increase in cardiovascular demand as the number of rounds continues in taekwondo athletes [1, 14, 32].

The results of our study showed a predominant estimated energy contribution of oxidative metabolism (65%) during simulated taekwondo combat, followed by ATP-PCr (27%) and glycolytic (8%) contribution. These results are in agreement with Campos et al. [1], who reported that energy contribution during a taekwondo combat simulation was predominantly supplied from aerobic sources, with lesser contribution from both the ATP-PCr and glycolytic energy systems. Furthermore, although caffeine supplementation had no effect on the ATP-PCr contribution, in the present study, there was an increase in ATP-PCr contribution in the third round in relation to the first and second rounds. This difference may be due to the method used to calculate ATP-PCr metabolism; a 1-min break between rounds is insufficient to determine VO_2_ offset, while the fast component of EPOC is considered for determination of energy contribution during the third round.

Ratings of perceived exertion increased linearly across rounds, although no differences were shown between caffeine and placebo. These results are in accordance with Santos et al. [14], who showed that caffeine supplementation did not reduce RPE during simulated taekwondo combat. However, Doherty and Smith [13] did show that caffeine ingestion reduced perceived exertion; 29% of the variance explaining the ergogenic effect of caffeine on performance was due to decreased RPE during the exercise. As opposed to endurance exercise, taekwondo combat is acyclic and athletes distribute the technical and tactical actions through the combat. Thus, our results showed that caffeine supplementation does not affect RPE during simulated taekwondo combat.

Caffeine ingestion resulted in increased peak lactate concentration following simulated taekwondo combat. This result is supported by other studies, which showed an increase in blood lactate concentration after caffeine ingestion [14, 17, 33]. For example, Santos et al. [14] showed that caffeine ingestion resulted in increased lactate concentrations during simulated taekwondo combat when compared to placebo. Furthermore, Lopes-Silva et al. [16] showed that caffeine ingestion following a 5-day weight loss period resulted in increased lactate concentrations after successive bouts of the Special Judo Fitness Test. Thus, the increase in the lactate concentration after caffeine ingestion corroborates with the results reported in the literature.

Our results showed that caffeine ingestion increased estimated glycolytic contribution during simulated taekwondo combat when compared to placebo. Although no study in the literature has reported that CAF supplementation increases glycolytic contribution during a combat taekwondo simulation, it is well established that caffeine increases anaerobic contribution during cycling exercise [16, 28]. Santos et al. [28] showed that caffeine ingestion increased anaerobic contribution during a 4-km cycling time-trial and two mechanisms have been proposed to explain the increase in blood lactate concentration. Firstly, CAF may increase anaerobic activity through its antagonistic action on peripheral adenosine receptors, which could prevent the inhibitory effects of adenosine on phosphofructokinase activity in skeletal muscle [34]. Secondly, caffeine may promote catecholamine release to facilitate the conversion of phosphorylase *b* to its more active *a* form, accelerating muscle glycogenolysis [33]. Although previous studies showed a decrease in glycolytic contribution over subsequent rounds during simulated taekwondo combat [1], our data show that caffeine supplementation actually increased estimated glycolytic contribution during simulated taekwondo combat simulation in relation to placebo. However, this did not translate across into any changes in technical actions performed by the athletes.

Caffeine supplementation did not affect post-exercise parasympathetic reactivation indexes after the taekwondo combat simulation. In contrast to our results, Bunsawat et al. [9] demonstrated that HR was increased 2-min after incremental treadmill exercise with caffeine in relation to placebo. However, this delay in HR was accompanied by an increase in time to exhaustion after caffeine supplementation. Thus, the delay in autonomic recovery after incremental exercise could have been caused by a combination of caffeine-induced sympathetic activation along with increased performance. Furthermore, although parasympathetic indexes after repeated sprint exercise have been correlated with anaerobic contribution, possibly through a high elevation of adrenergic factors and local metabolites during recovery [4], the increase in glycolytic metabolism after caffeine ingestion did not influence the parasympathetic indexes after simulated taekwondo combat.

## Practical Applications

Although time-motion performance variables during simulated taekwondo combat were not changed following supplementation, the results of the present study suggest that caffeine supplementation might be an effective strategy to increase glycolytic energy metabolism in taekwondo athletes, and could be an effective pharmacological strategy during training sessions to improve anaerobic capacity.

## Conclusion

In summary, caffeine supplementation increased estimated glycolytic metabolism contribution during simulated taekwondo combat, although this did not translate into improvements in performance. Furthermore, caffeine supplementation did not influence post-exercise parasympathetic reactivation following a taekwondo simulation.

## References

[1] Campos FA, Bertuzzi R, Dourado AC, Santos VGF, Franchini E. Energy demands in taekwondo athletes during combat simulation. Eur J Appl Physiol 2012; 112:1221–1228. 10.1007/s00421-011-2071-4 21769736

[2] Bridge CA, Ferreira da Silva Santos J, Chaabène H, Pieter W, Franchini E. Physical and physiological profiles of taekwondo athletes. Sports Med. 2014; 44:713–733. 10.1007/s40279-014-0159-9 24549477

[3] Santana J, Diefenthaeler F, Dal Pupo J, Detanico D, Guglielmo LGA, Santos SG. Anaerobic evaluation of Taekwondo athletes. Int Sportmed J 2014; 15: 492.

[4] Buchheit M, Papelier Y, Laursen PB, Ahmaidi S. Parasympathetic reactivation after repeated sprint exercise. Am J Physiol Heart Circ Physiol 2007; 293: H133–41. 17337589 10.1152/ajpheart.00062.2007

[5] Goldberger JJ, Le FK, Lahiri M, Kannankeril PJ, Ng J, Kadish AH. Assessment of parasympathetic reactivation after exercise. Am J Physiol Heart Circ Physiol 2006; 290: H2446–52. 16415073 10.1152/ajpheart.01118.2005

[6] Cole CR, Blackstone EH, Pashkow FJ, Snader CE, Lauer MS. Heart rate recovery immediately after exercise as a predictor of mortality N Engl J Med 1999; 28: 1351–1357. 10.1056/NEJM199910283411804 10536127

[7] Buchheit M, Papelier Y, Laursen PB, Ahmaidi S. Noninvasive assessment of cardiac parasympathetic function: postexercise heart rate recovery or heart rate variability? Am J Physiol Heart Circ Physiol 2007; 293: H8–10. 17384128 10.1152/ajpheart.00335.2007

[8] Pierpont GL, Stolpman DR, Gornick CC. Heart rate recovery postexercise as an index of parasympathetic activity. J Auton Nerv Syst 2000; 80:169–174. 10785283 10.1016/s0165-1838(00)00090-4

[9] Bunsawat K, White DW, Kappus RM, Baynard T. Caffeine delays autonomic recovery following acute exercise. Eur J Prev Cardiol 2014; 8 pii: 2047487314554867. 10.1177/2047487314554867 25297344

[10] Graham TE. Caffeine and exercise: Metabolism, endurance and performance. Sports Med 2001; 31: 785–807. 11583104 10.2165/00007256-200131110-00002

[11] Davis JK, Green JM. Caffeine and anaerobic performance: Ergogenic value and mechanisms of action. Sports Med 2009; 39: 813–832. 10.2165/11317770-000000000-00000 19757860

[12] Davis JM, Zhao Z, Stock HS, Mehl KA, Buggy J, Hand GA. Central nervous system effects of caffeine and adenosine on fatigue. Am J Physiol Regul Integr Comp Physiol 2003; 284: R399–404 12399249 10.1152/ajpregu.00386.2002

[13] Doherty M, Smith PM. Effects of caffeine ingestion on rating of perceived exertion during and after exercise: A meta-analysis. Scand J Med Sci Sports 2005; 15: 69–78. 15773860 10.1111/j.1600-0838.2005.00445.x

[14] Santos VGF, Santos VRF, Felippe LJC, Almeida JW Jr, Bertuzzi R, Kiss MAPD, et al Caffeine Reduces Reaction Time and Improves Performance in Simulated-Contest of Taekwondo. Nutrients 2014; 6: 637–49. 10.3390/nu6020637 24518826 PMC3942723

[15] Pallarés JG, Fernández-Elías VE, Ortega JF, Muñoz G, Muñoz-Guerra J, Mora-Rodríguez R. Neuromuscular responses to incremental caffeine doses: performance and side effects. Med Sci Sports Exerc 2013; 45: 2184–92. 23669879 10.1249/MSS.0b013e31829a6672

[16] Silva-Cavalcante MD, Correia-Oliveira CR, Santos RA, Lopes-Silva JP, Lima HM, Bertuzzi R, et al Caffeine increases anaerobic work and restores cycling performance following a protocol designed to lower endogenous carbohydrate availability. PLoS One 2013, 8, e72025 10.1371/journal.pone.0072025 23977198 PMC3747083

[17] Lopes-Silva JP, Felippe LJ, Silva-Cavalcante MD, Bertuzzi R, Lima-Silva AE. Caffeine ingestion after rapid weight loss in judo athletes reduces perceived effort and increases plasma lactate concentration without improving performance. Nutrients 2014; 6: 2931–2945. 10.3390/nu6072931 25054553 PMC4113770

[18] Vecchio FB, Franchini E, Vecchio AHM, Pieter W. Energy absorbed by electronic body protector from kicks in a taekwondo competition. Biol Sport 2011; 28: 75–78.

[19] Beneke R, Beyer T, Jachner C, Erasmus J, Hütler M. Energetics of karate kumite. Eur J Appl Physiol 2004; 92: 518–23. 15138826 10.1007/s00421-004-1073-x

[20] Borg GA. Psychophysical bases of perceived exertion. Med Sci Sports Exerc 1982; 14: 377–81. 7154893

[21] Hausswirth C, Bigard AX, Le Chevalier JM. The cosmed K4 telemetry system as an accurate device for oxygen uptake measurements during exercise. Int J Sports Med 1997; 18: 449–453. 9351691 10.1055/s-2007-972662

[22] Bertuzzi RCM, Franchini E, Kokubun E, Kiss MAPDM. Energy system contributions in indoor rock climbing. Eur J Appl Physiol 2007; 101: 293–300. 17602238 10.1007/s00421-007-0501-0

[23] Franchini E, Sterkowicz S, Szmatlan-Gabrys U, Gabrys T, Garnys M. Energy system contributions to the Special Judo Fitness Test. Int J Sports Physiol Perform 2011; 6: 334–343. 21911859 10.1123/ijspp.6.3.334

[24] di Prampero PE, Ferretti G. The energetics of anaerobic muscle metabolism: a reappraisal of older and recent concepts. Respir Physiol 1999; 118: 103–15. 10647856 10.1016/s0034-5687(99)00083-3

[25] Gastin P. Energy system interaction and relative contribution during maximal exercise. Sports Med 2011; 31: 725–741. 10.2165/00007256-200131100-00003 11547894

[26] Santos VG, Franchini E, Lima-Silva AE. Relationship between attack and skipping in taekwondo contests. J Strength Cond Res 2011; 25: 1743–1751. 21512402 10.1519/JSC.0b013e3181ddfb0f

[27] Cohen J. Statistical power analysis for the behavioural sciences. New York: Academic Press, 1977: 24–27.

[28] Santos RA, Kiss MA, Silva-Cavalcante MD, Correia-Oliveira CR, Bertuzzi R, Bishop DJ, et al Caffeine Alters Anaerobic Distribution and Pacing during a 4000-m Cycling Time Trial. PLoS One 2013, 8, e75399 10.1371/journal.pone.0075399 24058684 PMC3776790

[29] Crowe MJ, Leicht AS, Spinks WL. Physiological and cognitive responses to caffeine during repeated, high-intensity exercise. Int J Sport Nutr Exerc Metab 2006; 16: 528–544. 17240784 10.1123/ijsnem.16.5.528

[30] Aedma M, Timpmann S, Ööpik V. Effect of caffeine on upper-body anaerobic performance in wrestlers in simulated competition-day conditions. Int J Sport Nutr Exerc Metab 2013; 23: 601–9. 23751521 10.1123/ijsnem.23.6.601

[31] Felippe LC, Lopes-Silva JP, Bertuzzi R, McGinley C, Lima-Silva AE. Separate and Combined Effects of Caffeine and Sodium Bicarbonate Intake on Judo Performance. Int J Sports Physiol Perform 2015; 13. [Epub ahead of print] 10.1123/ijspp.2015-0020 26182440

[32] Bridge CA, Jones MA, Drust B. Physiological responses and perceived exertion during international taekwondo competition. Int J Sports Physiol Perfor 2009; 4: 485–93. 10.1123/ijspp.4.4.485 20029099

[33] Collomp K, Ahmaidi S, Audran M, Chanal JL, Préfaut C. Effects of caffeine ingestion on performance and anaerobic metabolism during the Wingate test. Int J Sports Med 1991; 12: 439–443. 1752708 10.1055/s-2007-1024710

[34] Simmonds MJ, Minahan CL. Caffeine improves supramaximal cycling but not the rate of anaerobic energy release. Eur J Appl Physiol 2010; 109: 287–95. 10.1007/s00421-009-1351-8 20082092

