# Correction: Caffeine Ingestion Increases Estimated Glycolytic Metabolism during Taekwondo Combat Simulation but Does Not Improve Performance or Parasympathetic Reactivation

**DOI:** 10.1371/journal.pone.0144849

**Published:** 2015-12-15

**Authors:** The PLOS ONE Staff

There are errors in the first sentence of the last paragraph of the Introduction, which were introduced during the typesetting process. The publisher apologizes for these errors. The correct sentence is: Therefore, the aim of this study was to evaluate the effect of caffeine ingestion on estimated energy systems contribution during simulated taekwondo combat and its subsequent effects on performance.
