# Correction: Caffeine Ingestion Increases Estimated Glycolytic Metabolism during Taekwondo Combat Simulation but Does Not Improve Performance or Parasympathetic Reactivation

**DOI:** 10.1371/journal.pone.0165350

**Published:** 2016-10-20

**Authors:** João Paulo Lopes-Silva, Jonatas Ferreira da Silva Santos, Braulio Henrique Magnani Branco, César Cavinato Cal Abad, Luana Farias de Oliveira, Irineu Loturco, Emerson Franchini

The following funding information is missing from the Funding section: Emerson Franchini was supported by FAPESP grant number: 2015/22315-9.
